# Supplementary material for: Smooth Interpolating Curves with Local Control and Monotone Alternating Curvature
Source: Comput Graph Forum. 2022 Oct 6;41(5):25–38. doi: 10.1111/cgf.14600 (PMC9827861; doi:10.1111/cgf.14600)
Supplement: Supplementary file 1 — Supplement Material [file CGF-41-25-s001.zip › Local-Smooth-Interpolating-MonoCurvature/extern/clothoids/docs/api-cpp/program_listing_file_Clothoids_PolyLine.hxx.html]

Program Listing for File PolyLine.hxx — Clothoids v2.0.9

### Navigation

- index
- toc
- Clothoids »
- Program Listing for File PolyLine.hxx

# Program Listing for File PolyLine.hxx¶

↰ Return to documentation for file (`Clothoids/PolyLine.hxx`)

```
/*--------------------------------------------------------------------------*\
 |                                                                          |
 |  Copyright (C) 2017                                                      |
 |                                                                          |
 |         , __                 , __                                        |
 |        /|/  \               /|/  \                                       |
 |         | __/ _   ,_         | __/ _   ,_                                |
 |         |   \|/  /  |  |   | |   \|/  /  |  |   |                        |
 |         |(__/|__/   |_/ \_/|/|(__/|__/   |_/ \_/|/                       |
 |                           /|                   /|                        |
 |                           \|                   \|                        |
 |                                                                          |
 |      Enrico Bertolazzi                                                   |
 |      Dipartimento di Ingegneria Industriale                              |
 |      Universita` degli Studi di Trento                                   |
 |      email: enrico.bertolazzi@unitn.it                                   |
 |                                                                          |
\*--------------------------------------------------------------------------*/


namespace G2lib {

  /*\
   |  ____       _       _     _
   | |  _ \ ___ | |_   _| |   (_)_ __   ___
   | | |_) / _ \| | | | | |   | | '_ \ / _ \
   | |  __/ (_) | | |_| | |___| | | | |  __/
   | |_|   \___/|_|\__, |_____|_|_| |_|\___|
   |               |___/
  \*/

  class CircleArc;
  class Biarc;
  class BiarcList;
  class ClothoidCurve;
  class ClothoidList;

  class PolyLine : public BaseCurve {
    friend class ClothoidList;
    friend class BiarcList;
  private:
    vector<LineSegment> m_polylineList;
    vector<real_type>   m_s0;
    real_type           m_xe;
    real_type           m_ye;

    mutable Utils::BinarySearch<int_type> m_lastInterval;

    mutable bool     m_aabb_done;
    mutable AABBtree m_aabb_tree;

    #ifndef DOXYGEN_SHOULD_SKIP_THIS
    class Collision_list {
      PolyLine const * pPL1;
      PolyLine const * pPL2;
    public:
      Collision_list( PolyLine const * _pPL1, PolyLine const * _pPL2 )
      : pPL1(_pPL1)
      , pPL2(_pPL2)
      {}

      bool
      operator () ( BBox::PtrBBox ptr1, BBox::PtrBBox ptr2 ) const {
        LineSegment const & LS1 = pPL1->m_polylineList[size_t(ptr1->Ipos())];
        LineSegment const & LS2 = pPL2->m_polylineList[size_t(ptr2->Ipos())];
        return LS1.collision( LS2 );
      }
    };
    #endif

    void
    resetLastInterval() {
      bool ok;
      int_type & lastInterval = *m_lastInterval.search( std::this_thread::get_id(), ok );
      lastInterval = 0;
    }

  public:

    //explicit
    PolyLine()
    : BaseCurve(G2LIB_POLYLINE)
    , m_aabb_done(false)
    { this->resetLastInterval(); }

    void
    init();

    void
    copy( PolyLine const & l );

    //explicit
    PolyLine( PolyLine const & PL )
    : BaseCurve(G2LIB_POLYLINE)
    , m_aabb_done(false)
    { this->resetLastInterval(); copy(PL); }

    int_type
    findAtS( real_type & s ) const;

    explicit PolyLine( LineSegment const & LS );
    explicit PolyLine( CircleArc const & C, real_type tol );
    explicit PolyLine( Biarc const & B, real_type tol );
    explicit PolyLine( ClothoidCurve const & B, real_type tol );
    explicit PolyLine( ClothoidList const & B, real_type tol );
    explicit PolyLine( BaseCurve const & C );

    PolyLine const & operator = ( PolyLine const & s )
    { copy(s); return *this; }

    LineSegment const &
    getSegment( int_type n ) const;

    int_type
    numSegments() const
    { return int_type(m_polylineList.size()); }

    int_type
    numPoints() const
    { return int_type(m_s0.size()); }

    void polygon( real_type * x, real_type * y) const;
    void init( real_type x0, real_type y0 );
    void push_back( real_type x, real_type y );
    void push_back( LineSegment const & C );
    void push_back( CircleArc const & C, real_type tol );
    void push_back( Biarc const & C, real_type tol );
    void push_back( ClothoidCurve const & C, real_type tol );
    void push_back( ClothoidList const & L, real_type tol );

    void
    build(
      real_type const * x,
      real_type const * y,
      int_type npts
    );

    void build( LineSegment const & L );
    void build( CircleArc const & C, real_type tol );
    void build( Biarc const & B, real_type tol );
    void build( ClothoidCurve const & C, real_type tol );
    void build( ClothoidList const & CL, real_type tol );

    void
    bbox(
      real_type & xmin,
      real_type & ymin,
      real_type & xmax,
      real_type & ymax
    ) const override;

    void
    bbox_ISO(
      real_type   /* offs */,
      real_type & /* xmin */,
      real_type & /* ymin */,
      real_type & /* xmax */,
      real_type & /* ymax */
    ) const override {
      UTILS_ERROR0( "PolyLine::bbox( offs ... ) not available!\n" );
    }

    /*\
     |  _    _   _____    _                _
     | | |__| |_|_   _| _(_)__ _ _ _  __ _| |___
     | | '_ \ '_ \| || '_| / _` | ' \/ _` | / -_)
     | |_.__/_.__/|_||_| |_\__,_|_||_\__, |_\___|
     |                               |___/
    \*/

    void
    bbTriangles(
      std::vector<Triangle2D> & tvec,
      real_type                 max_angle = Utils::m_pi/6, // 30 degree
      real_type                 max_size  = 1e100,
      int_type                  icurve    = 0
    ) const override;

    void
    bbTriangles_ISO(
      real_type                 offs,
      std::vector<Triangle2D> & tvec,
      real_type                 max_angle = Utils::m_pi/6, // 30 degree
      real_type                 max_size  = 1e100,
      int_type                  icurve    = 0
    ) const override;

    void
    bbTriangles_SAE(
      real_type                 offs,
      std::vector<Triangle2D> & tvec,
      real_type                 max_angle = Utils::m_pi/6, // 30 degree
      real_type                 max_size  = 1e100,
      int_type                  icurve    = 0
    ) const override {
      this->bbTriangles_ISO( -offs, tvec, max_angle, max_size, icurve );
    }

    real_type
    length() const override
    { return m_s0.back(); }

    real_type
    length_ISO( real_type ) const override {
      UTILS_ERROR0( "PolyLine::length( offs ) not available!\n" );
      return 0;
    }

    real_type
    xBegin() const override
    { return m_polylineList.front().xBegin(); }

    real_type
    yBegin() const override
    { return m_polylineList.front().yBegin(); }

    real_type
    xEnd() const override
    { return m_polylineList.back().xEnd(); }

    real_type
    yEnd() const override
    { return m_polylineList.back().yEnd(); }

    real_type
    X( real_type s ) const override {
      int_type idx = this->findAtS( s );
      real_type ss = m_s0[size_t(idx)];
      return m_polylineList[size_t(idx)].X(s-ss);
    }

    real_type
    X_D( real_type s ) const override {
      int_type idx = this->findAtS( s );
      return m_polylineList[size_t(idx)].m_c0;
    }

    real_type
    X_DD( real_type ) const override
    { return 0; }

    real_type
    X_DDD( real_type ) const override
    { return 0; }

    real_type
    Y( real_type s ) const override {
      int_type idx = this->findAtS( s );
      real_type ss = m_s0[size_t(idx)];
      return m_polylineList[size_t(idx)].Y(s-ss);
    }

    real_type
    Y_D( real_type s ) const override {
      int_type idx = this->findAtS( s );
      return m_polylineList[size_t(idx)].m_s0;
    }

    real_type
    Y_DD( real_type ) const override
    { return 0; }

    real_type
    Y_DDD( real_type ) const override
    { return 0; }

    real_type
    theta( real_type s ) const override;

    real_type
    theta_D( real_type s ) const override;

    real_type
    theta_DD( real_type s ) const override;

    real_type
    theta_DDD( real_type s ) const override;

    void
    eval(
      real_type   s,
      real_type & x,
      real_type & y
    ) const override {
      int_type idx = this->findAtS( s );
      real_type ss = m_s0[size_t(idx)];
      m_polylineList[size_t(idx)].eval( s-ss, x, y );
    }

    void
    eval_D(
      real_type   s,
      real_type & x_D,
      real_type & y_D
    ) const override {
      int_type idx = this->findAtS( s );
      real_type ss = m_s0[size_t(idx)];
      m_polylineList[size_t(idx)].eval_D( s-ss, x_D, y_D );
    }

    void
    eval_DD(
      real_type,
      real_type & x_DD,
      real_type & y_DD
    ) const override
    { x_DD = y_DD = 0; }

    void
    eval_DDD(
      real_type,
      real_type & x_DDD,
      real_type & y_DDD
    ) const override
    { x_DDD = y_DDD = 0; }

    // ---

    void
    eval_ISO(
      real_type   s,
      real_type   offs,
      real_type & x,
      real_type & y
    ) const override {
      int_type idx = this->findAtS( s );
      real_type ss = m_s0[size_t(idx)];
      m_polylineList[size_t(idx)].eval_ISO( s-ss, offs, x, y );
    }

    void
    eval_ISO_D(
      real_type   s,
      real_type   offs,
      real_type & x_D,
      real_type & y_D
    ) const override {
      int_type idx = this->findAtS( s );
      real_type ss = m_s0[size_t(idx)];
      m_polylineList[size_t(idx)].eval_ISO_D( s-ss, offs, x_D, y_D );
    }

    void
    eval_ISO_DD(
      real_type,
      real_type,
      real_type & x_DD,
      real_type & y_DD
    ) const override
    { x_DD = y_DD = 0; }

    void
    eval_ISO_DDD(
      real_type,
      real_type,
      real_type & x_DDD,
      real_type & y_DDD
    ) const override
    { x_DDD = y_DDD = 0; }

    /*\
     |  _                        __
     | | |_ _ __ __ _ _ __  ___ / _| ___  _ __ _ __ ___
     | | __| '__/ _` | '_ \/ __| |_ / _ \| '__| '_ ` _ \
     | | |_| | | (_| | | | \__ \  _| (_) | |  | | | | | |
     |  \__|_|  \__,_|_| |_|___/_|  \___/|_|  |_| |_| |_|
    \*/

    void
    translate( real_type tx, real_type ty ) override {
      std::vector<LineSegment>::iterator il;
      for ( il = m_polylineList.begin(); il != m_polylineList.end(); ++il )
        il->translate( tx, ty );
    }

    void
    rotate(
      real_type angle,
      real_type cx,
      real_type cy
    ) override {
      std::vector<LineSegment>::iterator il;
      for ( il = m_polylineList.begin(); il != m_polylineList.end(); ++il )
        il->rotate( angle, cx, cy );
    }

    void
    reverse() override;

    void
    scale( real_type sc ) override;

    void
    changeOrigin( real_type newx0, real_type newy0 ) override;

    void
    trim( real_type s_begin, real_type s_end ) override;

    void
    trim( real_type s_begin, real_type s_end, PolyLine & newPL ) const;

    int_type
    closestPoint_ISO(
      real_type   x,
      real_type   y,
      real_type & X,
      real_type & Y,
      real_type & S,
      real_type & T,
      real_type & DST
    ) const override;

    int_type
    closestPoint_ISO(
      real_type   /* x    */,
      real_type   /* y    */,
      real_type   /* offs */,
      real_type & /* X    */,
      real_type & /* Y    */,
      real_type & /* S    */,
      real_type & /* T    */,
      real_type & /* DST  */
    ) const override {
      UTILS_ERROR( "PolyLine::closestPoint( ... offs ... ) not available!\n" );
    }

    /*\
     |             _ _ _     _
     |    ___ ___ | | (_)___(_) ___  _ __
     |   / __/ _ \| | | / __| |/ _ \| '_ \
     |  | (_| (_) | | | \__ \ | (_) | | | |
     |   \___\___/|_|_|_|___/_|\___/|_| |_|
    \*/

    bool
    collision( PolyLine const & C ) const;

    bool
    collision_ISO(
      real_type        offs,
      PolyLine const & CL,
      real_type        offs_CL
    ) const {
      UTILS_ASSERT0(
        Utils::isZero(offs) && Utils::isZero(offs_CL),
        "PolyLine::collision( offs ... ) not available!\n"
      );
      return this->collision( CL );
    }

    /*\
     |   _       _                          _
     |  (_)_ __ | |_ ___ _ __ ___  ___  ___| |_
     |  | | '_ \| __/ _ \ '__/ __|/ _ \/ __| __|
     |  | | | | | ||  __/ |  \__ \  __/ (__| |_
     |  |_|_| |_|\__\___|_|  |___/\___|\___|\__|
    \*/

    void
    intersect(
      PolyLine const    & pl,
      vector<real_type> & ss0,
      vector<real_type> & ss1
    ) const;

    void
    intersect(
      PolyLine const & pl,
      IntersectList  & ilist,
      bool             swap_s_vals
    ) const;

    void
    intersect_ISO(
      real_type        offs,
      PolyLine const & pl,
      real_type        offs_pl,
      IntersectList  & ilist,
      bool             swap_s_vals
    ) {
      UTILS_ASSERT0(
        Utils::isZero(offs) && Utils::isZero(offs_pl),
        "PolyLine::intersect( offs ... ) not available!\n"
      );
      this->intersect( pl, ilist, swap_s_vals );
    }

    void
    info( ostream_type & stream ) const override
    { stream << "PolyLine\n" << *this << '\n'; }

    friend
    ostream_type &
    operator << ( ostream_type & stream, PolyLine const & P );

    void
    build_AABBtree( AABBtree & aabb ) const;

    void
    build_AABBtree() const {
      if ( !m_aabb_done ) {
        this->build_AABBtree( m_aabb_tree );
        m_aabb_done = true;
      }
    }

  };

}
```

### Quick search

### Table of Contents

- Matlab Interface Manual
- C++ API
- MATLAB API

«
hide menu

menu
sidebar
»

### Navigation

- index
- toc
- Clothoids »
- Program Listing for File PolyLine.hxx

© Copyright 2021, Enrico Bertolazzi and Marco Frego.
Created using Sphinx 4.2.0.
